# Effects of Acute *Ecklonia cava* Supplementation on Submaximal Cycling and Repeated-Sprint Performance in Trained Cyclists: A Randomised Double-Blind Placebo-Controlled Crossover Trial

**DOI:** 10.3390/nu18203430

**Published:** 2026-10-19

**Authors:** Yunus Ali, Tom Gurney, James Brouner, Owen Spendiff

**Affiliations:** 1 Division of Surgery & Interventional Science, University College London, London WC1E 6BT, UK; yunus.ali.19@ucl.ac.uk; 2 Department of Sport and Health Sciences, University of Exeter, Exeter EX4 4PY, UK; 3 School of Life Sciences, Kingston University, Kingston upon Thames KT1 2EE, UK; james.brouner@kingston.ac.uk (J.B.); o.spendiff@kingston.ac.uk (O.S.)

**Keywords:** nutrition, algae, cycling, performance

## Abstract

Background/Objectives: This study examined whether acute *Ecklonia cava* (EC) ingestion altered physiological responses during submaximal cycling or power output during subsequent repeated maximal sprints in experienced male cyclists. Methods: Twelve cyclists completed a randomised, double-blind, placebo-controlled crossover trial. Participants ingested 263 mg of EC extract or placebo 30 min before 60 min of cycling at 55% of the maximum work rate, followed by three 20 s maximal sprints. Blood glucose, blood lactate, heart rate, oxygen uptake and respiratory exchange ratio (RER) were measured during submaximal cycling; peak power, mean power, blood glucose and blood lactate were assessed during the repeated-sprint test. Outcomes were analysed using linear mixed-effects models incorporating treatment, time, treatment × time, experimental period and allocated sequence. Results: Blood glucose was higher overall following EC than placebo (4.23 ± 0.23 vs. 4.02 ± 0.33 mmol·L^−1^; mean difference, 0.213 mmol·L^−1^; 95% CI, 0.046 to 0.379; *p* = 0.023), but the treatment × time interaction was not significant (*p* = 0.939). EC did not affect submaximal blood lactate, heart rate, oxygen uptake or RER (all *p* ≥ 0.096). EC did not alter peak power (mean difference, −2.58 W; 95% CI, −29.51 to 24.35; *p* = 0.832), mean power (5.93 W; 95% CI, −2.47 to 14.33; *p* = 0.167), post-sprint glucose (*p* = 0.481) or post-sprint lactate (*p* = 0.060). No treatment × time interaction was identified for any repeated-sprint outcome (all *p* ≥ 0.267). Conclusions: Acute ingestion of 263 mg of EC extract produced a modest overall elevation in blood glucose during submaximal cycling, but did not improve repeated-sprint performance or materially alter the other measured physiological responses. These findings do not support an acute ergogenic effect of EC in experienced male cyclists under the conditions tested.

## 1. Introduction

*Ecklonia cava* (EC) is an edible brown alga rich in phlorotannins, a class of marine polyphenols that includes eckol, dieckol, bieckol, and phlorofucofuroeckol derivatives [1]. EC extracts and isolated phlorotannins have demonstrated antioxidant, anti-inflammatory, metabolic, and vascular activity across a range of experimental models [1,2]. These properties have prompted interest in whether acute EC ingestion could modify physiological responses to exercise and enhance physical performance.

The vascular, metabolic, and redox-modulating properties attributed to EC provide a rationale for investigating it as an acute pre-exercise ergogenic aid. These actions could be particularly relevant to sustained endurance cycling and the maintenance of performance during repeated maximal sprints. EC extract and its phlorotannin dieckol have promoted nitric-oxide-mediated vasodilation through calcium signalling and the PI3K/Akt/eNOS pathway in cellular and zebrafish models [2]. A comparable response in humans could increase peripheral perfusion and support oxygen and substrate delivery during prolonged exercise. Improved perfusion may also facilitate recovery between repeated maximal efforts, for which the restoration of energy availability is important. Although these vascular actions have not been confirmed during human exercise, they provide a biologically plausible pathway through which acute EC ingestion could influence cycling performance.

EC may also influence glucose regulation. In adults with prediabetes, a single 600 mg dose of a polyphenol-rich EC extract reduced the postprandial glucose response without significantly affecting plasma insulin [3]. This finding demonstrates that EC can acutely modify glucose handling in humans, although the response during exercise may differ from that observed after a meal in a clinical population. During prolonged cycling, the maintenance of circulating glucose contributes to substrate availability, while the capacity to generate repeated maximal power may be affected by the metabolic demands of the preceding exercise. Modulation of glucose availability therefore represents a further potential pathway through which EC could influence both submaximal physiological responses and subsequent sprint performance.

The phlorotannins present in EC may additionally modify the redox response to exercise. Excessive reactive oxygen species production has been associated with contractile dysfunction and muscular fatigue [4], while polyphenol supplementation has been investigated as a potential means of supporting exercise performance and recovery. A systematic review and meta-analysis reported a small overall improvement in exercise performance following polyphenol supplementation, although the included interventions predominantly involved supplementation for at least seven days and compounds other than EC [5]. Reactive oxygen species also contribute to normal cellular signalling; therefore, antioxidant activity should not be considered inherently ergogenic [6]. Nevertheless, the combined vascular, metabolic, and redox properties attributed to EC provide a coherent rationale for examining its acute effects during exercise. In the present study, these pathways were considered potential explanations for any observed responses rather than directly measured mechanisms.

Published evidence concerning EC and human exercise performance appears limited to one acute investigation. The study administered a 72 mg EC polyphenol drink or placebo to 20 male college students 30 min before a treadmill protocol comprising 30 min of submaximal exercise followed by an incremental test to exhaustion [7]. EC increased time to exhaustion by 2.39 min. Mean maximal oxygen uptake was 6.5% higher following EC, although the difference was not statistically significant. Blood glucose measured 3 min after exhaustive exercise was 9.9% higher following EC, whereas the post-exercise blood lactate response showed a nonsignificant tendency to be lower. The authors proposed that altered glucose oxidation, lactate production, antioxidant activity, or circulation might have contributed to the performance response, while acknowledging that the underlying mechanism could not be determined [7]. These findings provide preliminary evidence of an acute ergogenic effect and a basis for investigation in other trained populations and exercise modalities.

The extent to which the findings of Oh et al. (2010) [7] apply to trained cyclists remains unknown because exercise modality, training status, protocol duration, and the selected performance outcome may influence the response to supplementation. The present study therefore examined acute EC supplementation in experienced male cyclists completing 60 min of submaximal cycling followed by three maximal 20 s cycling sprints. This protocol enabled the assessment of physiological responses during controlled, prolonged aerobic exercise and the subsequent capacity to generate repeated maximal power, representing two distinct demands relevant to cycling performance.

A 263 mg dose of commercial EC extract was selected as an exploratory dose higher than the 72 mg dose used by Oh et al. (2010) [7], while remaining within the adult safety limit assessed by the European Food Safety Authority [8]. The EFSA threshold provided a safety boundary for dose selection rather than evidence of ergogenic efficacy or dose optimality. Human pharmacokinetic data specific to EC phlorotannins remain limited, and evidence from other brown-algal phlorotannins and preclinical EC models indicates that systemic availability and metabolism may vary according to the source, compound, and experimental model [9,10]. Administration 30 min before exercise was therefore selected to replicate the timing used by Oh et al. (2010) [7], enabling a comparison with the previous human exercise study rather than being presented as an established optimal period for skeletal-muscle exposure.

Accordingly, this study aimed to determine whether ingesting 263 mg of EC extract 30 min before exercise influenced blood glucose, blood lactate, heart rate, oxygen uptake, or the respiratory exchange ratio during 60 min of submaximal cycling and whether it affected power output during subsequent repeated maximal sprints in experienced male cyclists. Based on the higher post-exercise glucose concentration, tendency towards lower blood lactate, and improved time to exhaustion reported by Oh et al. (2010) [7], it was hypothesised that EC would help maintain circulating glucose availability, attenuate metabolic and cardiorespiratory disturbance during submaximal cycling, and preserve power output during the repeated-sprint test. The study therefore tested whether the acute physiological and ergogenic effects previously reported following EC ingestion could be reproduced in a trained cycling population using a higher dose and a cycling-specific exercise protocol.

## 2. Materials and Methods

### 2.1. Study Design

This study used a randomised, double-blind, placebo-controlled crossover protocol to investigate the acute effects of EC supplementation on physiological responses during submaximal cycling and performance during a repeated-sprint performance test (RSPT). The study was conducted at the laboratories of Kingston University, London, where participants attended on four separate visits. Visit 1 comprised anthropometric measurements and an incremental cycling test to determine maximal oxygen consumption (V̇O_2max_) and maximum work rate (WR_max_). During Visit 2, participants completed the full exercise protocol for familiarisation, and Visits 3 and 4 were the experimental trials in which participants received EC or placebo in a randomised order. The experimental trials were separated by a seven-day washout period. The full exercise protocol involved a 60 min submaximal cycling session at 55% of WR_max_, followed by a 10 min recovery period and the RSPT.

**Figure 1:**
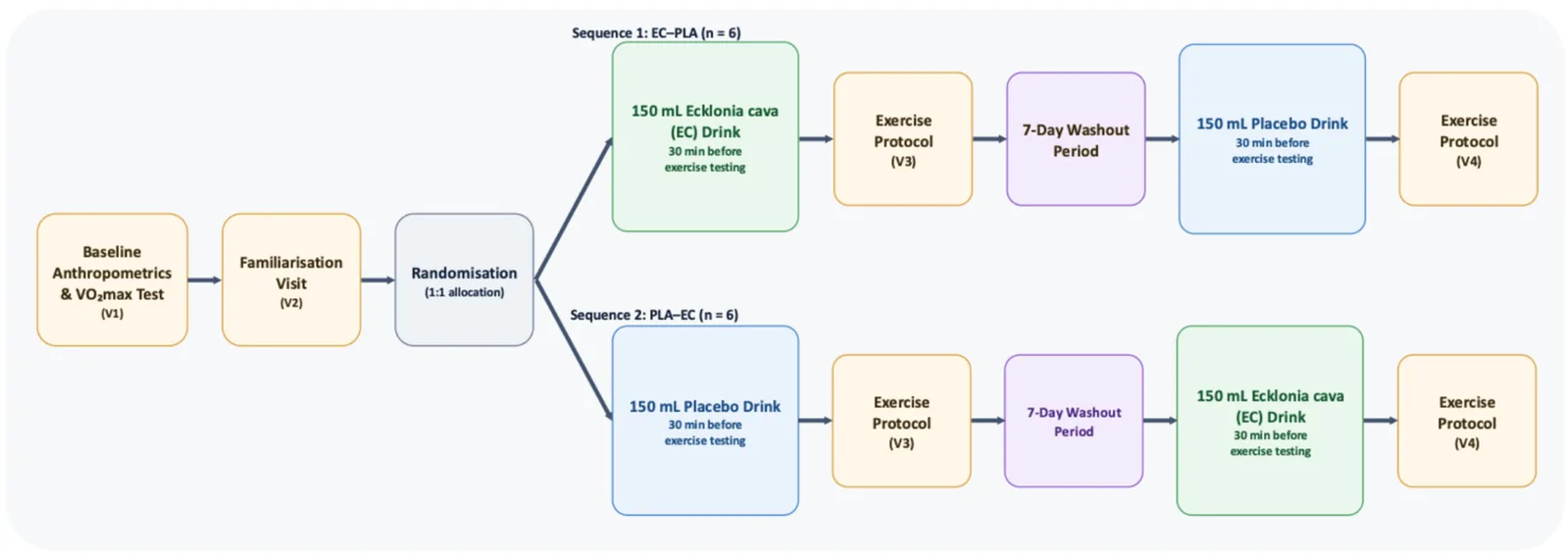
Schematic of the study design.

The study protocol is shown in Figure 1. Participant progression is presented in the CONSORT flow diagram (available in Supplementary Files—Figure S1). Reporting was guided by the CONSORT 2025 and the PRESENT 2020 checklist.

Ethical approval was granted by the Kingston University Research Ethics Committee (Ethics ID: 1501). All participants provided written informed consent, and the study was conducted in accordance with the Declaration of Helsinki.

### 2.2. Participants

Twelve trained male cyclists completed the study (mean ± SD; age, 25 ± 2 years; body mass index, 21.3 ± 2.0 kg·m^−2^; and V̇O_2max_, 50.0 ± 8.4 mL·kg^−1^·min^−1^). Participants reported cycling 180 ± 52 km and completing 8 ± 2 h of cycling training per week.

Participants were eligible if they were aged 18–50 years and had completed at least four hours of cycling training per week for a minimum of two years. No minimum V̇O_2max_ threshold was used. Exclusion criteria were current smoking, a history of cardiovascular disease or metabolic disease, musculoskeletal injury and the use of antihypertensive medication. Participants completed a Physical Activity Readiness Questionnaire before enrolment. Recruitment was open to cyclists of both sexes; however, only males enrolled.

An a priori sample-size calculation was performed using G*Power (Version 3.1) for the primary performance outcomes of peak and mean power during the repeated-sprint test. An F-test (“ANOVA: repeated measure, within factors”) was used to approximate the within-participant crossover design. The calculation specified one group, six repeated measurements representing three sprints under each experimental condition, Cohen’s f = 0.30, a =0.05, 80% power, a correlation of 0.60 among repeated measurements and a non-sphericity correction of ε = 1.00. The assumed medium effect size was informed by previous research demonstrating meaningful improvements in exercise performance following acute *Ecklonia cava* supplementation, including enhanced time-to-exhaustion performance reported by Oh et al. [7], as directly applicable data on acute *Ecklonia cava* supplementation and repeated-sprint performance was unavailable. The required sample size was eleven participants. Twelve participants completed both experimental conditions and were included in the analyses.

### 2.3. Randomisation and Blinding

An independent laboratory technician generated the allocation sequence using a computer-based random-number generator in Microsoft Excel. Participants were allocated in a 1:1 ratio to receive EC–placebo or placebo–EC, with six participants assigned to each sequence.

The technician retained the allocation sequence and treatment codes; therefore, participants and the researcher responsible for enrolment, testing and outcome assessment had no access to treatment assignments during data collection. The treatment code was released after the final participant had completed data collection.

After completing both experimental trials, participants completed a questionnaire assessing whether they could distinguish between the drinks, which condition they believed to have received first and whether they experienced any adverse effects.

### 2.4. Experimental Drinks

All drinks were prepared in the laboratories of Kingston University, and each had a total volume of 150 mL. The placebo consisted of sugar-free cordial (Robinsons Squash, Britvic PLC, Hemel Hempstead, UK) diluted with water at a ratio of 1:4. The EC drink contained the same cordial solution with the addition of 263 mg of a commercially manufactured standardised powdered alcohol extract of *Ecklonia cava* (SeaPolynol™ [SEANOL-P]; Botamedi Inc., Jeju, Republic of Korea). Drinks were prepared and coded by an independent laboratory technician and provided in identical opaque bottles.

According to the regulatory specification evaluated by the European Food Safety Authority, SeaPolynol contains 90 ± 5% phlorotannins, determined using the Folin–Ciocalteu method and expressed as phloroglucinol equivalents, including 6.6–9.9% dieckol [8]. The administered dose therefore comprised 263 mg of the total EC extract, rather than 263 mg of pure phlorotannins. Based on the central value of the regulatory specification, this would correspond nominally to approximately 237 mg of phlorotannin equivalents. The administered extract was not independently chemically analysed; therefore, these compositional values represent the standardised product specification rather than direct measurements of the study batch.

### 2.5. Supplementation Protocol and Washout Period

The 263 mg dose of EC was selected with reference to the EFSA safety assessment, which identified 263 mg/day as the maximum daily intake considered safe for adults [8]. This represented a higher nominal extract dose than the 72 mg administered by Oh et al. [7] and was selected as an exploratory upper safety-based dose. The EFSA assessment addressed safety rather than efficacy; therefore, 263 mg was not considered an established optimal ergogenic dose. At each experimental visit, participants consumed the entire assigned drink, after which the 30 min pre-exercise period commenced. Participants remained seated during this period. This administration period was selected to replicate the acute protocol used by Oh et al. [7]; it was not assumed to represent an established optimal period for systemic or skeletal-muscle exposure. Experimental visits were separated by a seven-day washout period to minimise potential carryover between conditions. Participants received the alternative intervention during the subsequent experimental visit.

### 2.6. Baseline Measurements and Incremental Cycling Test (Visit 1)

Anthropometric measurements including body mass (kg) and stature (cm) were recorded during the first visit and at the beginning of each subsequent visit. A 20 μL capillary blood sample was collected from the fingertip to measure glucose and lactate concentrations using a Biosen C-Line Sport analyser (EKF Diagnostics GmbH, Barleben, Germany).

All cycling tests were performed on a mechanically braked cycle ergometer (Monark Exercise AB, Varberg, Sweden). Saddle height was adjusted for each participant during Visit 1 and recorded and reproduced at subsequent visits.

Respiratory variables were measured using a Vyntus CPX metabolic system (Vyaire Medical GmbH, Bavaria, Germany), and heart rate was monitored using a Polar F10 monitor (Polar Electro Oy, Kempele, Finland). The incremental cycling test began with a 3 min warm-up at 0 W and 60–70 revolutions per minute (rpm). The test commenced at 120 W, with work rate continuously increasing at 25 W·min^−1^ until volitional exhaustion or inability to maintain 80 rpm. Respiratory variables were recorded continuously and averaged over 15 s intervals. V̇O_2_max was defined as the highest 15 s V̇O_2_ value recorded before test termination. Maximal effort was assessed using the criteria described by Beltz et al.: a plateau in VO_2_ with an increase of ≤150 mL·min^−1^, despite an increase in work rate, a peak heart rate within ±10 bpm of the age-predicted maximum (220—age), an RER ≥ 1.15, and an RPE ≥ 17 on the Borg 6–20 scale [1,11]. WR_max_ was defined as the highest work rate attained during the incremental test and was recorded to the nearest completed 15 s stage. The workload for the submaximal trials was calculated as 55% of each participant’s WR_max_ and applied manually to the ergometer.

### 2.7. Familiarisation (Visit 2) and Pre-Trial Standardisation

During Visit 2, anthropometric data were recorded again, and participants completed the full exercise protocol without supplementation. This visit familiarised participants with the ergometer, respiratory mask, cadence requirements and sprint procedures.

Participants were instructed to maintain their usual training throughout the study. Participants completed a seven-day activity log and a three-day dietary record before the familiarisation visit and were instructed to replicate the behaviours recorded before the subsequent visits. Participants were instructed to avoid additional supplements and strenuous exercise for 24 h before testing. All visits were conducted at approximately the same time of day.

### 2.8. Submaximal Cycling Test

Participants cycled for 60 min at 55% of their individual WR_max_, calculated from Visit 1, while maintaining a cadence of 80 rpm. The work rate remained constant throughout the test. Respiratory variables and heart rate were recorded continuously. Respiratory data were averaged over 15 s intervals, after which respiratory and heart-rate values were averaged over three consecutive 20 min periods: 0–20, 20–40 and 40–60 min. A 20 μL capillary blood sample was collected at rest and at 20, 40 and 60 min for measurement of glucose and lactate concentrations.

### 2.9. Repeated-Sprint Performance Test

After completing the submaximal test, participants rested for 10 min before undertaking the repeated-sprint performance test. Participants completed a 5 min warm-up at 50 W, including two 3 s preparatory sprints, followed by 1 min of rest. Participants then performed three 20 s maximal sprints from a flying start. Resistance was set at 8.5% of body mass and applied when cadence exceeded 90 rpm.

Each sprint was followed by 4 min of active recovery at 50 W. During the second minute of each recovery period, a 20 μL fingertip capillary blood sample was collected for measurement of glucose and lactate concentrations.

**Figure 2:**
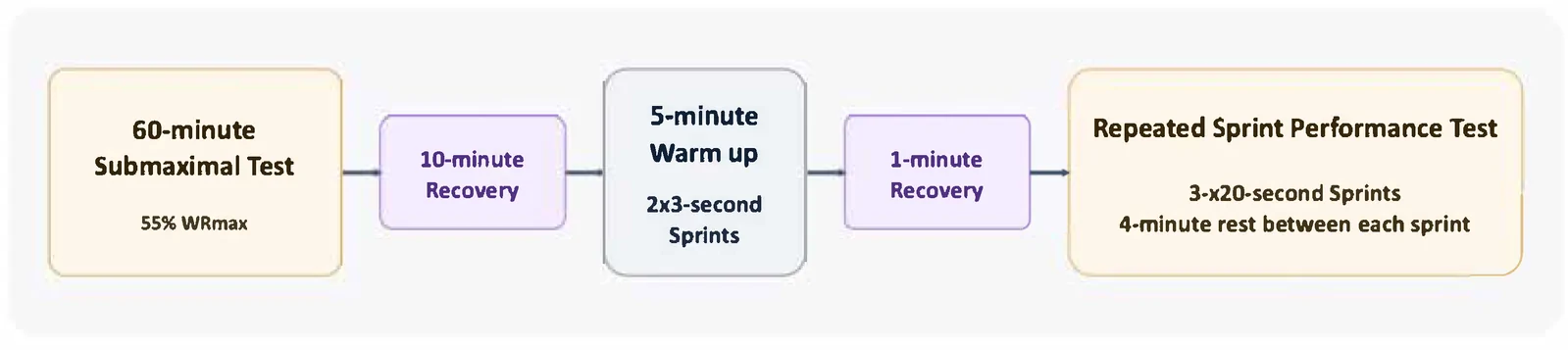
Schematic of the exercise protocol.

Peak and mean power were recorded using Monark Peak Bike software and expressed as absolute values (W) and relative to body mass (W·kg^−1^). The exercise protocol is shown in Figure 2.

### 2.10. Statistical Analysis

Statistical analyses were conducted using IBM SPSS Statistics for macOS (version 29.0.2.0; IBM Corp., Armonk, NY, USA). Data are presented as mean ± SD, unless otherwise stated. Each outcome was analysed using a linear mixed-effects model with treatment, time, treatment × time, experimental period, and allocated sequence as fixed effects. Time was treated as categorical, participant was included as a random intercept, and a compound-symmetry covariance structure was specified for repeated measurements within each treatment condition. Models were estimated using restricted maximum likelihood, and fixed effects were evaluated using Type III tests.

Model assumptions were assessed using residual histograms, normal Q–Q plots, residual-versus-predicted plots, and Shapiro–Wilk tests. Submaximal blood lactate, peak power, and mean power were natural-log-transformed because of non-normally distributed residuals; sensitivity analyses using untransformed data produced the same conclusions. Participant-level condition means were used to calculate paired mean differences, 95% CIs, and Cohen’s d_z_, defined as the mean paired difference divided by the SD of the paired differences. Descriptive statistics and effect sizes for transformed outcomes are presented on the original scale. All tests were two-sided, with statistical significance set at *p* < 0.05.

## 3. Results

### 3.1. Submaximal Exercise Responses

**Figure 3:**
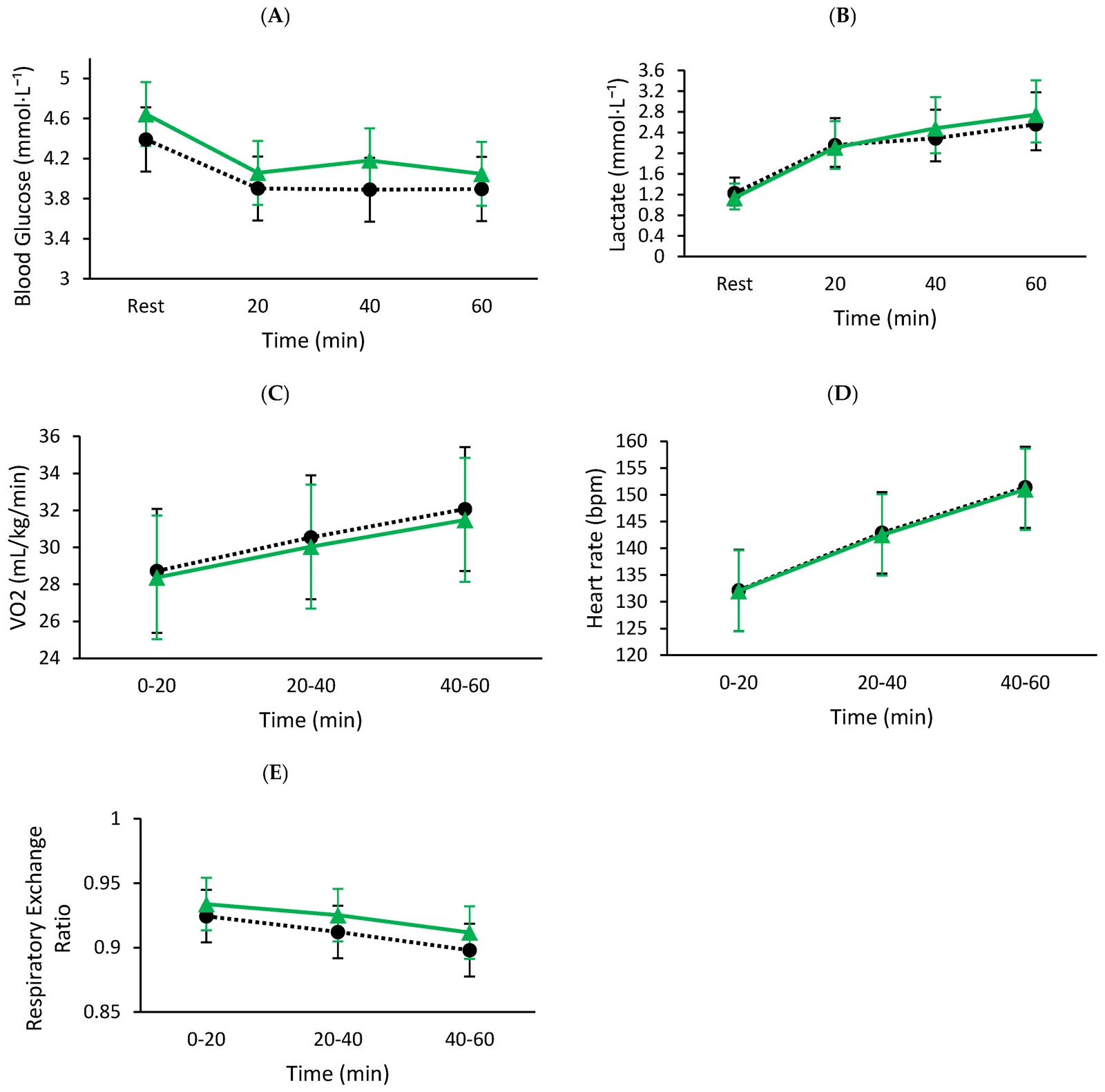
Physiological responses during 60 min of submaximal cycling following *Ecklonia cava* and placebo ingestion. Estimated marginal means and 95% confidence intervals are presented for (**A**) blood glucose, (**B**) blood lactate, (**C**) oxygen uptake, (**D**) heart rate and (**E**) respiratory exchange ratio. Blood glucose and lactate were measured at rest and after 20, 40 and 60 min; heart rate, oxygen uptake and respiratory exchange ratio were averaged across the 0–20, 20–40 and 40–60 min intervals. Blood lactate estimates and confidence intervals were back-transformed following analysis on the logarithmic scale. EC, *Ecklonia cava*; PLA, placebo; RER, respiratory exchange ratio.

Submaximal exercise responses are presented in Table 1 and Figure 3. A significant main effect of treatment was identified for blood glucose, with higher concentrations following EC than placebo, *p* = 0.023. The participant-level paired mean difference was 0.213 mmol·L^−1^ (95% CI 0.046 to 0.379), with a standardised within-participant effect of d_z_ = 0.81 (95% CI 0.14 to 1.46). Blood glucose also changed significantly over time (*p* < 0.001), but the treatment × time interaction was not significant (*p* = 0.939).

No significant treatment effects were identified for blood lactate, heart rate, VO_2_, or RER, all *p* ≥ 0.096. Each outcome changed significantly over time (all *p* < 0.001); however, none demonstrated a significant treatment × time interaction, all *p* ≥ 0.810. No significant period or sequence effects were identified for any submaximal outcome (all *p* ≥ 0.181).

### 3.2. Repeated-Sprint Performance Test

Repeated-sprint performance test results are presented in Table 2. No significant treatment effects were identified for peak power, mean power, blood glucose, or blood lactate (all *p* ≥ 0.060). Similarly, no treatment × time interactions were identified for any repeated-sprint outcome (all *p* ≥ 0.267).

Peak power (*p* = 0.031), mean power (*p* < 0.001), and blood lactate (*p* < 0.001) changed significantly across the repeated-sprint protocol, whereas blood glucose did not (*p* = 0.114). A significant period effect was identified for blood lactate (*p* = 0.042). No other period effects were identified (all (*p* ≥ 0.213)), and no significant sequence effects were identified for any repeated-sprint outcome (all (*p* ≥ 0.095)).

### 3.3. Model Diagnostics

Visual examination of residual histograms, normal Q-Q plots, and residual-versus-predicted plots identified no substantial violations of model assumptions following transformation (available in Supplementary Files—Figures S2 and S3). Residuals from the models for submaximal blood lactate, peak power, and mean power deviated from normality on the original scale (Shapiro–Wilk *p* = 0.004, *p* = 0.005, and *p* = 0.009, respectively). Following natural-log transformation, no statistically significant departures from residual normality were identified (*p* = 0.072, *p* = 0.113, and *p* = 0.174, respectively). Sensitivity analyses using the untransformed outcomes produced the same inferential conclusions.

### 3.4. Exit Questionnaire

At study completion, 11 participants (91.7%) reported that they could not detect a difference in taste between the EC and placebo drinks, and 10 (83.3%) did not correctly identify the treatment received during their first experimental period. No adverse effects were reported.

## 4. Discussion

This study examined the acute effects of ingesting 263 mg of EC extract before 60 min of submaximal cycling and a subsequent repeated-sprint test in experienced male cyclists. EC produced a modest overall elevation in blood glucose during submaximal exercise, with concentrations 0.213 mmol·L^−1^ higher than placebo (95% CI: 0.046 to 0.379 mmol·L^−1^; Cohen’s d_z_ = 0.81). However, the absence of a treatment × time interaction indicates that the pattern of glucose change during exercise did not differ between conditions. EC did not affect blood lactate, heart rate, oxygen uptake or the respiratory exchange ratio (RER) during submaximal cycling and did not improve peak or mean power during the repeated-sprint test. Collectively, these findings do not support an acute ergogenic effect of EC under the conditions tested.

The higher overall glucose concentration following EC is partially consistent with Oh et al., who reported 9.9% higher blood glucose 3 min after exhaustive exercise following acute EC polyphenol ingestion [7]. The present finding should nevertheless be interpreted cautiously. The treatment × time interaction was not significant, meaning that the data do not demonstrate that EC prevented the exercise-associated decline in glucose or exerted a distinct effect at a particular sampling point. Furthermore, the absolute difference was small, and its practical significance for exercise performance remains uncertain. Although EC could conceivably affect glucose regulation, hepatic glucose output, muscle glucose uptake, insulin and catecholamine concentrations were not measured. The present data therefore cannot establish whether the glucose difference resulted from altered glucose production, utilisation, distribution or another process.

Importantly, the higher glucose concentration was not accompanied by improved repeated-sprint performance. The estimated EC–placebo difference was −2.58 W for peak power (95% CI: −29.51 to 24.35 W) and 5.93 W for mean power (95% CI: −2.47 to 14.33 W). Both confidence intervals included zero, and no treatment × time interaction was detected. Circulating glucose during submaximal exercise is therefore a physiological response rather than direct evidence of improved performance. The present results show that the observed glucose difference did not translate into a measurable improvement in subsequent sprint power.

The performance findings differ from those of Oh et al. [7], who reported a 2.39 min increase in treadmill time to exhaustion after ingestion of 72 mg of EC polyphenols. However, the studies differed in several important respects. Oh et al. [7] studied male college students completing treadmill exercise followed by an incremental test to exhaustion, whereas the present study recruited experienced cyclists and used 60 min of constant-load cycling followed by three maximal 20 s sprints. The interventions also differed in dose and possibly in extract composition. Time-to-exhaustion tests and repeated-sprint protocols assess different dimensions of exercise capacity and may respond differently to supplementation. Consequently, the present findings did not reproduce the performance benefit reported by Oh et al. [7] under the experimental conditions used here, but the available evidence cannot identify whether population, exercise modality, protocol, dose, extract composition or another methodological difference explains the divergent performance outcomes.

Heart rate and oxygen uptake increased during the 60 min cycling bout, but their responses were nearly identical between EC and placebo, with no treatment × time interactions. The progressive increases therefore reflect the physiological response to prolonged exercise rather than an effect of EC. An upward drift in heart rate during constant-load cycling is consistent with cardiovascular drift [12]. A concurrent increase in oxygen uptake can also occur if cycling efficiency decreases as exercise continues. Previous research has demonstrated reductions in gross efficiency and increases in muscle oxygen uptake during prolonged constant-load cycling, including in trained cyclists [13]. Potential contributors include thermoregulatory strain, changes in hydration and progressive reductions in mechanical or metabolic efficiency [12]. As core temperature, plasma volume and gross cycling efficiency were not measured, the cause of the drift observed in the present study cannot be determined. Nevertheless, its comparable magnitude in both conditions provides no evidence that EC altered this response.

EC also had no discernible effect on submaximal blood lactate or RER. The absence of treatment effects or treatment × time interactions does not support the original hypotheses that acute EC ingestion would attenuate lactate accumulation or substantially alter substrate utilisation during the cycling bout. These variables should not be interpreted as performance measures. Rather, they provide physiological context for a fixed-workload exercise protocol in which the external workload was identical between conditions.

During the repeated-sprint test, neither post-sprint glucose nor lactate differed significantly between treatments. Mean glucose concentrations were approximately 4 mmol·L^−1^ in both conditions, and mean lactate concentrations were 8.62 mmol·L^−1^ after EC and 8.17 mmol·L^−1^ after placebo. The latter values indicate substantial lactate accumulation rather than an absence of glycolytic demand. Blood samples were obtained during the second minute of each recovery period and therefore represent early recovery values rather than the maximal metabolic response to each sprint. Blood lactate may continue to rise during the first several minutes after maximal cycling [14], meaning that this sampling schedule may not have captured peak concentrations. The treatment estimate for sprint lactate was 0.458 mmol·L^−1^ (95% CI: −0.105 to 1.022 mmol·L^−1^; *p* = 0.060). This interval includes no effect as well as a potentially moderate increase and should not be interpreted as evidence that EC altered lactate production or clearance.

A significant period effect was identified for sprint lactate, with concentrations approximately 0.5 mmol·L^−1^ higher during the first experimental period irrespective of treatment. Because treatment order and experimental period were incorporated into the mixed model, the estimated treatment effect was adjusted for this visit-related difference. The physiological basis of the period effect is uncertain and should not be attributed to familiarisation or another specific mechanism without supporting measurements. No period effect was observed for the other sprint outcomes, and no sequence effects were detected. However, the absence of a significant sequence effect does not definitively exclude carryover.

Several biological properties of EC have been proposed as potentially relevant to exercise, including effects on redox regulation, vascular function and glucose metabolism. These mechanisms remain plausible research questions but were not evaluated in this study. Oh et al. proposed that free-radical-scavenging and procirculatory actions might have contributed to their findings, while acknowledging that their study did not establish a mechanism. Similarly, evidence that ingestion alters plasma antioxidant capacity does not demonstrate that active compounds reach skeletal muscle or improve exercise performance [15]. Antioxidant activity is not synonymous with an ergogenic effect, and reactive oxygen species also have important physiological signalling functions. As oxidative-stress biomarkers, antioxidant capacity, nitric oxide, blood flow and inflammatory markers were not measured, the present findings cannot confirm or refute any of these proposed pathways.

Furthermore, the 263 mg EC extract dose was selected as an exploratory dose within the adult safety limit assessed by the European Food Safety Authority rather than as an established optimal ergogenic dose. Administration 30 min before exercise was selected to reproduce the timing used by Oh et al. [7]. Available pharmacokinetic evidence does not establish that this interval produces optimal circulating concentrations or biologically active exposure within skeletal muscle [9,10]. The present findings therefore apply specifically to the formulation, dose and administration interval examined and should not be extrapolated to alternative acute or longer-term supplementation protocols.

### Limitations

The sample comprised only 12 male cyclists, limiting statistical precision and generalisability to women, less-trained individuals and other athletic populations. Confidence intervals for several outcomes remained compatible with potentially meaningful effects in either direction; therefore, non-significant findings should not be interpreted as proof of equivalence. The assessment of several outcomes without a prospectively designated primary outcome also makes the glucose finding exploratory and in need of independent confirmation. The EC extract was standardised under its regulatory specification to contain 90 ± 5% phlorotannins, expressed as phloroglucinol equivalents, including 6.6–9.9% dieckol [8]. Thus, the 263 mg dose represented total EC extract and corresponded nominally to approximately 237 mg of phlorotannin equivalents. Although independent batch-specific analysis was not performed, future studies would benefit from profiling individual phlorotannins to facilitate a comparison between formulations. Furthermore, no pharmacokinetic, vascular, antioxidant, inflammatory or oxidative-stress measurements were obtained, preventing evaluation of the proposed mechanisms. Capillary glucose and lactate concentrations were collected at predetermined intervals and may not have captured transient or peak responses, particularly following the maximal sprints. Finally, the findings apply only to a single acute dose administered 30 min before exercise and cannot establish the effects of repeated supplementation, alternative doses or different administration periods.

## 5. Conclusions

Acute ingestion of 263 mg of *Ecklonia cava* extract 30 min before exercise produced a modest overall elevation in blood glucose during submaximal cycling but did not alter the pattern of glucose change over time. EC also had no discernible effect on blood lactate, heart rate, oxygen uptake, RER, or peak and mean power during the subsequent repeated-sprint test. These findings therefore do not support an acute ergogenic effect of EC in experienced male cyclists under the specific conditions tested.

The present results did not reproduce the performance improvement reported by Oh et al.; however, differences in participants, extract composition, dose, exercise modality and performance protocols prevent identification of the factor responsible for the contrasting findings. Further research should evaluate chemically characterised EC extracts using prospectively defined performance outcomes, larger sex-inclusive samples and pharmacokinetic or mechanistic measurements. Alternative doses, administration intervals and longer supplementation periods also warrant investigation.

## Figures and Tables

**Table 1:** Physiological responses during submaximal exercise following *Ecklonia cava* and placebo ingestion.

Variable	EC, Mean ± SD	Placebo, Mean ± SD	Paired Mean Difference (95% CI)	Cohen’s d_z_ (95% CI)	Treatment (*p*)	Time (*p*)	Treatment × Time (*p*)	Period (*p*)	Sequence (*p*)
Blood glucose (mmol·L^−1^)	4.23 ± 0.23	4.02 ± 0.33	0.213 (0.046 to 0.379)	0.81 (0.14 to 1.46)	0.023 *	<0.001 *	0.939	0.884	0.964
Blood lactate (mmol·L^−1^) †	2.21 ± 0.48	2.20 ± 0.66	0.009 (−0.321 to 0.339)	0.02 (−0.55 to 0.58)	0.955	<0.001 *	0.810	0.802	0.181
Heart rate (beats·min^−1^)	141.9 ± 11.6	142.1 ± 10.2	−0.29 (−4.38 to 3.80)	−0.05 (−0.61 to 0.52)	0.883	<0.001 *	0.991	0.689	0.982
VO_2_ (mL·kg^−1^·min^−1^)	30.0 ± 4.9	30.4 ± 5.3	−0.48 (−2.11 to 1.15)	−0.19 (−0.75 to 0.39)	0.528	<0.001 *	0.945	0.295	0.403
RER	0.924 ± 0.029	0.912 ± 0.026	0.012 (−0.002 to 0.026)	0.55 (−0.07 to 1.15)	0.096	<0.001 *	0.898	0.686	0.890

Values are mean ± SD based on participant-level condition means. For blood glucose and blood lactate, condition means were calculated across rest and the 20, 40, and 60 min measurements. For heart rate, VO_2_, and RER, condition means were calculated across the three 20 min exercise intervals. Mean differences were calculated as EC minus placebo. Paired mean differences, 95% CIs, and Cohen’s d_z_ values were calculated from participant-level paired condition means. Fixed-effect (*p*) values were obtained from crossover-adjusted linear mixed-effects models that included treatment, time, treatment × time, period, and allocated sequence. * indicates significance (*p* < 0.05). EC, *Ecklonia cava;* RER, respiratory exchange ratio. † Natural-log-transformed before mixed-model analysis because of non-normally distributed residuals; descriptive statistics and standardised effect sizes are presented on the original scale.

**Table 2:** Repeated-sprint performance test following *Ecklonia cava* and placebo.

Variable	EC, Mean ± SD	Placebo, Mean ± SD	Paired Mean Difference (95% CI)	Cohen’s d_z_ (95% CI)	Treatment (*p*)	Time (*p*)	Treatment × Time (*p*)	Period (*p*)	Sequence (*p*)
Peak power (W) †	781.5 ± 190.2	784.1 ± 185.6	−2.58 (−29.51 to 24.35)	−0.06 (−0.63 to 0.51)	0.832	**0.031**	0.719	0.213	0.095
Mean power (W) †	604.1 ± 125.0	598.2 ± 123.8	5.93 (−2.47 to 14.33)	0.45 (−0.16 to 1.04)	0.167	**<0.001**	0.849	0.754	0.233
Blood lactate (mmol·L^−1^)	8.62 ± 2.09	8.17 ± 2.46	0.458 (−0.105 to 1.022)	0.52 (−0.10 to 1.11)	0.060	**<0.001**	0.267	**0.042**	0.359
Blood glucose (mmol·L^−1^)	4.12 ± 0.26	3.99 ± 0.54	0.125 (−0.236 to 0.487)	0.22 (−0.36 to 0.79)	0.481	0.114	0.306	0.725	0.444

Values are mean ± SD based on participant-level condition means across the repeated-sprint performance tests. Mean differences were calculated as EC minus placebo. Paired mean differences, 95% CIs, and Cohen’s d_z_ values were calculated from participant-level paired condition means. Fixed-effect (*p*) values were obtained from crossover-adjusted linear mixed-effects models that included treatment, time, treatment × time, period, and allocated sequence. Bold type indicates (*p* < 0.05). EC, *Ecklonia cava*. † Natural-log-transformed before mixed-model analysis because of non-normally distributed residuals; descriptive statistics and standardised effect sizes are presented on the original scale.

## Data Availability

The datasets generated and/or analysed during the current study are available from the corresponding author on request.

## Supplementary Materials

The following supporting information can be downloaded at https://www.mdpi.com/article/10.3390/nu18203430/s1, Figure S1: CONSORT 2025 Flow Diagram of Participant Progression; Figure S2: Graphical Analysis of Normality Testing for Repeated-sprint Performance Data; Figure S3: Graphical Analysis of Normality Testing for Submaximal Exercise Data.
