# Supplementary material for: Effects of Acute Ecklonia cava Supplementation on Submaximal Cycling and Repeated-Sprint Performance in Trained Cyclists: A Randomised Double-Blind Placebo-Controlled Crossover Trial
Source: Nutrients. 2026 Oct 19;18(20):3430. doi: 10.3390/nu18203430 (PMC13708020; doi:10.3390/nu18203430)
Supplement: Supplementary file 1 [file nutrients-18-03430-s001.zip › nutrients-4568802-supplementary.pdf]

Supplementary Figures

Effects of Acute Ecklonia cava Supplementation on Submaximal Cycling and Repeated-sprint Performance in Trained Cyclists

Figure S1: CONSORT 2025 Flow Diagram of Participant Progression

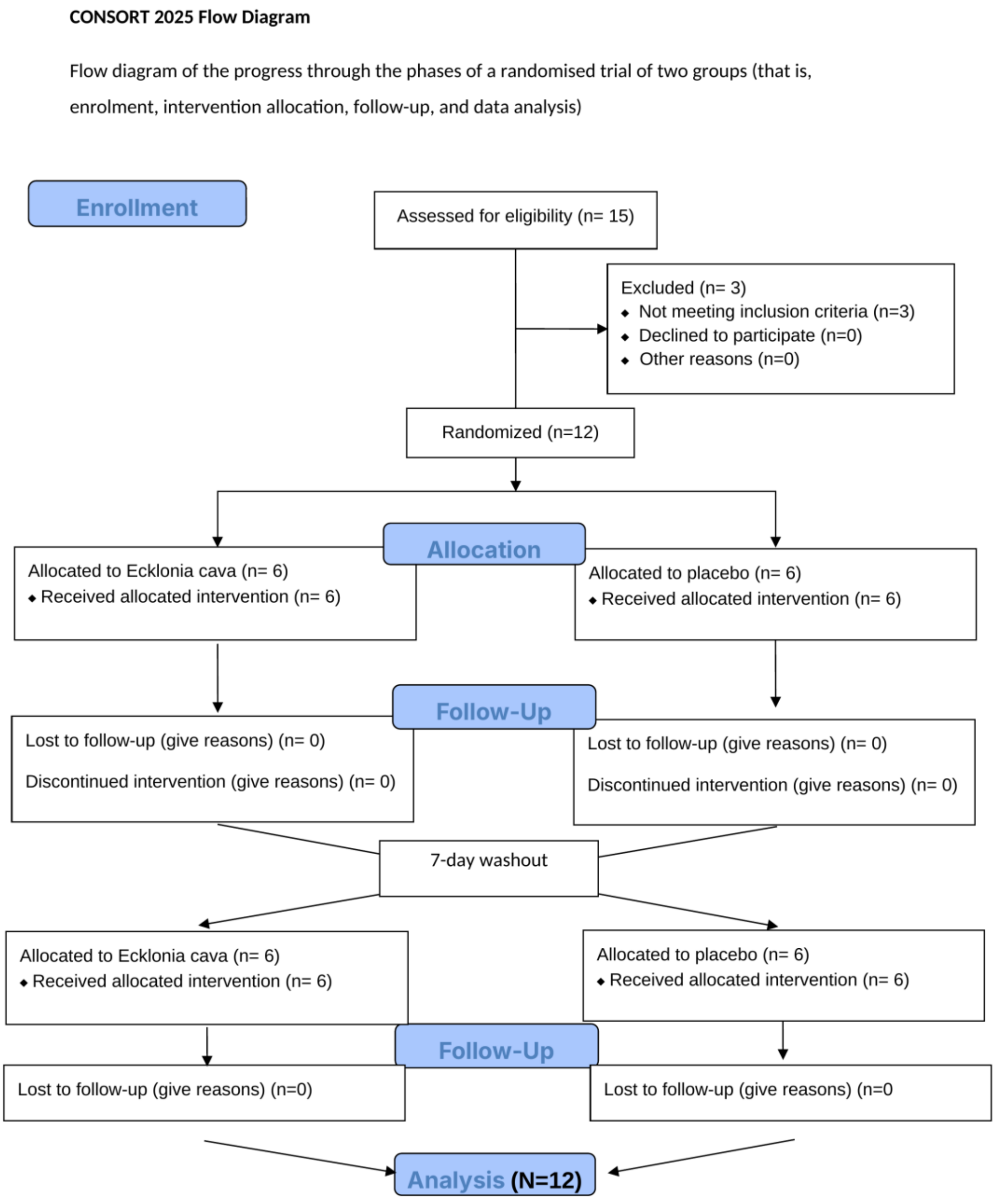

Figure S1. CONSORT 2025 flow diagram of the progress of participants through the phases of the randomised, double-blind, placebo-controlled two-period crossover trial (enrolment, allocation, follow-up and analysis for each supplementation period, separated by a 7-day washout).

Figure S2: Graphical Analysis of Normality Testing for Repeated-sprint Performance Data

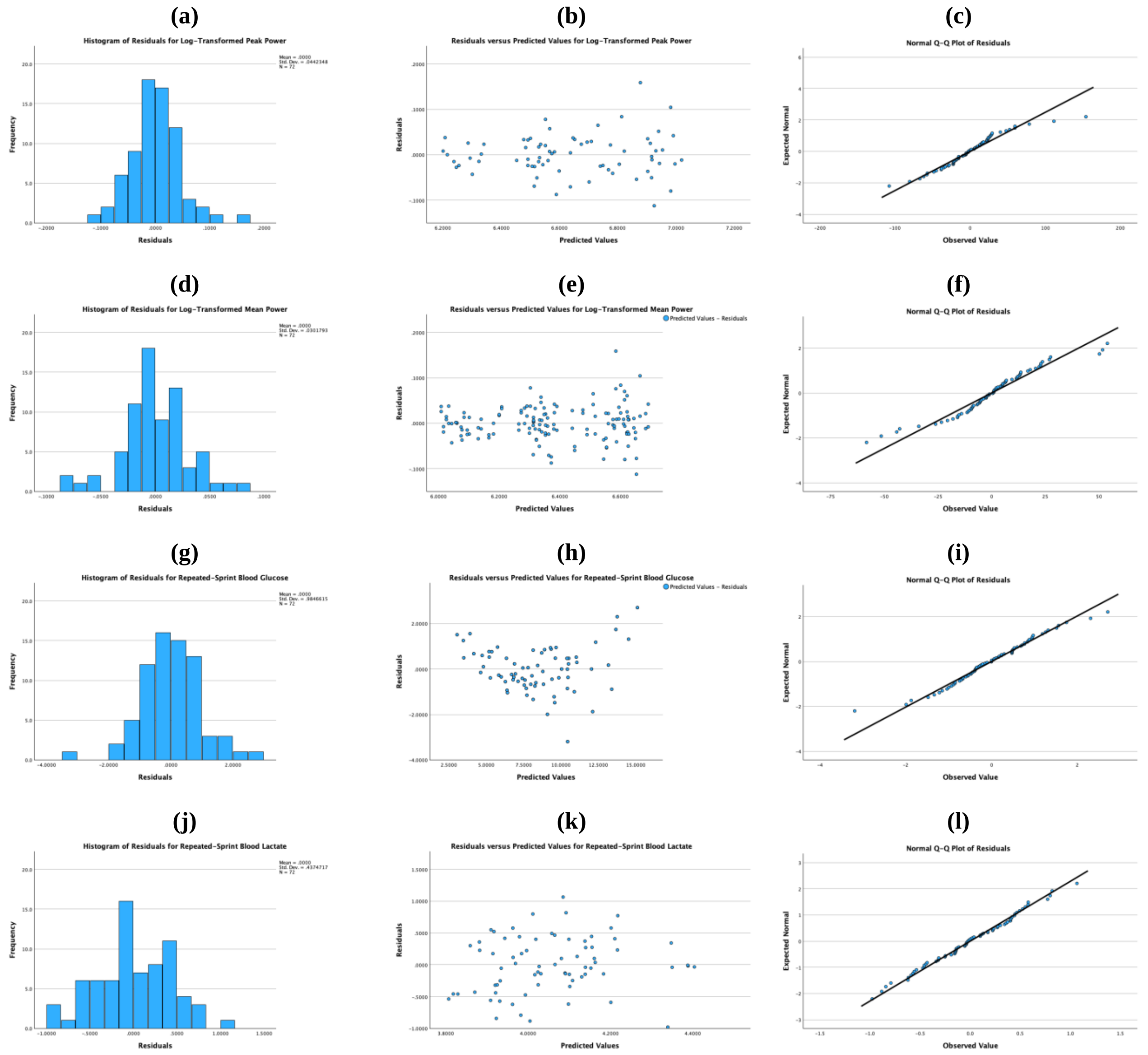

Figure S2. Graphical analysis of model residuals for repeated-sprint performance outcomes (linear mixed-effects models including treatment, time, treatment×time, period and sequence as fixed effects and participant as a random intercept). For each outcome, residual normality was assessed via a histogram of residuals, a plot of residuals against predicted values, and a normal Q–Q plot of residuals.

(a) Histogram of residuals for log-transformed peak power;
(b) Residuals versus predicted values for log-transformed peak power;
(c) Normal Q–Q plot of residuals for log-transformed peak power;

(d) Histogram of residuals for log-transformed mean (average) power;
(e) Residuals versus predicted values for log-transformed mean (average) power;
(f) Normal Q–Q plot of residuals for log-transformed mean (average) power;

(g) Histogram of residuals for repeated-sprint blood glucose;
(h) Residuals versus predicted values for repeated-sprint blood glucose;
(i) Normal Q–Q plot of residuals for repeated-sprint blood glucose;

(j) Histogram of residuals for repeated-sprint blood lactate;
(k) Residuals versus predicted values for repeated-sprint blood lactate;
(l) Normal Q–Q plot of residuals for repeated-sprint blood lactate.

Figure S3. Graphical Analysis of Normality Testing for Submaximal Exercise Data

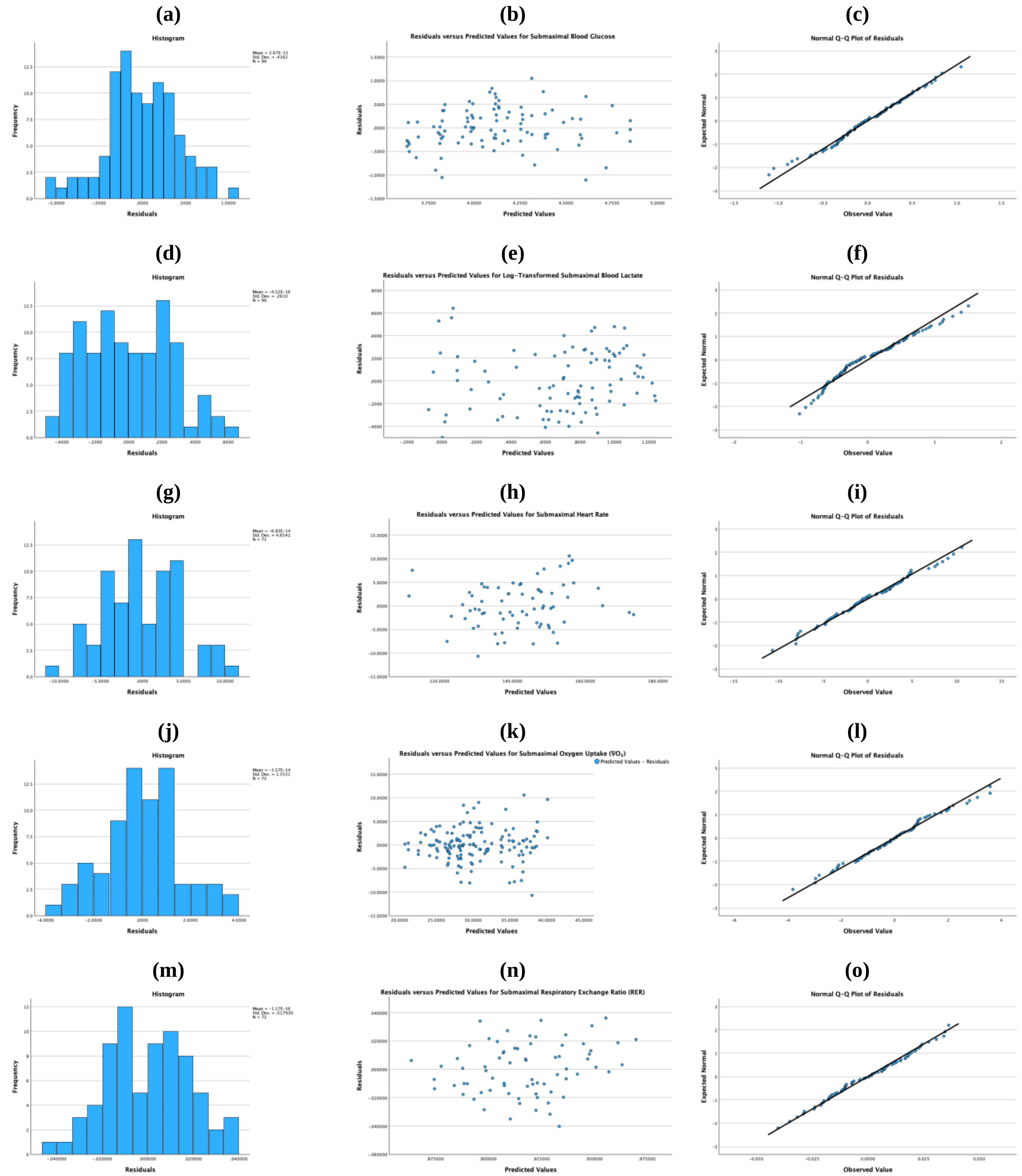

Figure S3. Graphical analysis of model residuals for submaximal exercise outcomes (linear mixed-effects models including treatment, time, treatment×time, period and sequence as fixed effects and participant as a random intercept). For each outcome, residual normality was assessed via a histogram of residuals, a plot of residuals against predicted values, and a normal Q–Q plot of residuals.

(a) Histogram of residuals for submaximal blood glucose;
(b) Residuals versus predicted values for submaximal blood glucose;
(c) Normal Q–Q plot of residuals for submaximal blood glucose;

(d) Histogram of residuals for log-transformed submaximal blood lactate;
(e) Residuals versus predicted values for log-transformed submaximal blood lactate;

(f) Normal Q–Q plot of residuals for log-transformed submaximal blood lactate;

(g) Histogram of residuals for submaximal heart rate;
(h) Residuals versus predicted values for submaximal heart rate;
(i) Normal Q–Q plot of residuals for submaximal heart rate;

(j) Histogram of residuals for submaximal oxygen uptake ($\dot{V}O_2$);
(k) Residuals versus predicted values for submaximal oxygen uptake ($\dot{V}O_2$);
(l) Normal Q–Q plot of residuals for submaximal oxygen uptake ($\dot{V}O_2$);

(m) Histogram of residuals for submaximal respiratory exchange ratio (RER);
(n) Residuals versus predicted values for submaximal respiratory exchange ratio (RER);
(o) Normal Q–Q plot of residuals for submaximal respiratory exchange ratio (RER).
